# Pre-diagnostic serum levels of EGFR and ErbB2 and genetic glioma risk variants: a nested case-control study

**DOI:** 10.1007/s13277-015-4742-y

**Published:** 2016-02-23

**Authors:** Florentin Späth, Ulrika Andersson, Anna M. Dahlin, Hilde Langseth, Eivind Hovig, Tom Børge Johannesen, Kjell Grankvist, Benny Björkblom, Carl Wibom, Beatrice Melin

**Affiliations:** 1 Department of Radiation Sciences, Umeå University, 901 87 Umeå, Sweden; 2 Department of Oncology, Umeå University, 901 87 Umeå, Sweden; 3 Computational Life Science Cluster (CLiC), Umeå University, 901 87 Umeå, Sweden; 4 Cancer Registry of Norway, Institute of Population‐based Cancer Research, Oslo, Norway; 5 Department of Informatics, University of Oslo, Oslo, Norway; 6 Department of Tumor Biology, Institute for Cancer Research, Oslo University Hospital, Oslo, Norway; 7 Institute for Cancer Genetics and Informatics, Oslo University Hospital, Oslo, Norway; 8 Department of Medical Biosciences, Clinical Chemistry, Umeå University, 901 85 Umeå, Sweden; 9 Department of Chemistry, Umeå University, 901 87 Umeå, Sweden

**Keywords:** Glioma, SNP, Serum EGFR, Serum ErbB2, Pre‐diagnostic

## Abstract

Genetic variants have been associated with the risk of developing glioma, but functional mechanisms on disease phenotypic traits remain to be investigated. One phenotypic trait of glioblastoma is the mutation and amplification of the epidermal growth factor receptor (*EGFR*) gene. We investigated associations between pre-diagnostic serum protein concentrations of EGFR and ErbB2, both members of the EGFR family, and future risk of glioma. Further, we studied if *EGFR* glioma risk variants were associated with EGFR and ErbB2 serum levels. We assessed the associations between genetic glioma risk variants and serum concentrations of EGFR and ErbB2, as measured in pre-diagnostic cohort serum samples of 593 glioma patients and 590 matched cancer-free controls. High serum EGFR and ErbB2 levels were associated with risk of developing glioblastoma (*P* = 0.008; OR = 1.58, 95 % CI = 1.13–2.22 and *P* = 0.017, OR = 1.63, 95 % CI = 1.09–2.44, respectively). High serum ErbB2 concentration was also associated with glioma risk overall (*P* = 0.049; OR = 1.39, 95 % CI = 1.00–1.93). Glioma risk variants were not associated with high serum protein abundance. In contrast, the *EGFR* risk variant rs4947986 (*T*) was correlated with decreased EGFR serum levels (study cohort *P* = 0.024 and controls *P* = 0.009). To our knowledge, this is the first study showing an association of EGFR and ErbB2 serum levels with glioma more than a decade before diagnosis, indicating that EGFR and ErbB2 serum proteins are important in early gliomagenesis. However, we did not find evidence that glioma risk variants were associated with high pre-diagnostic serum concentrations of EGFR and ErbB2.

**Electronic supplementary material:**

The online version of this article (doi:10.1007/s13277-015-4742-y) contains supplementary material, which is available to authorized users.

## Introduction

The incidence of gliomas is approximately six new diagnosed cases per 100,000 inhabitants per year [1]. The 5-year survival rate for patients with glioblastomas has improved with combined chemoradiotherapy, but still most patients are not long-term survivors [2]. Curative treatment is rare due to the infiltrative growth of the tumor [3]. General cancer risk factors, such as alcohol and smoking, have not been associated with brain tumors [4] and currently there is no sufficient knowledge of the etiology of gliomas. Established glioma risk factors are ionizing radiation, hereditary syndromes, and increasing age, whereas asthma and allergy are inversely associated with the risk of developing gliomas [5, 6]. Increasing evidence supports a genetic component in glioma etiology, including both observations of familiar aggregation of gliomas [7–9] and observations of nine single nucleotide polymorphisms (SNPs) being associated with risk of developing gliomas, identified through large scale genome-wide association studies (GWAS) [10–14] (Table 1). Additional polymorphisms suggested to be associated with glioma risk include rs4947979 and rs4947986 at 7p11.2 (*EGFR*), identified by our group through a candidate gene approach [15] (Table 1). In another study, rs4947986 was associated with poor survival, although failing replication in a validation dataset [16].

**Table 1:** Genetic variants associated with glioma

SNP^a^	Locus	Gene	Reference
**rs4295627**	8q24.21	*CCDC26*	[10]
**rs498872**	11q23.3	*PHLDB1*	[10]
**rs6010620**	20q13.33	*RTEL1*	[10]
**rs4977756**	9p21.3	*CDKN2B-AS1*	[10]
**rs1412829**	9p21.3	*CDKN2B-AS1*	[11]
**rs2252586**	7p11.2	*EGFR*	[12]
**rs11979158**	7p11.2	*EGFR*	[12]
**rs78378222**	17p13.1	*TP53*	[13, 14]
rs4947979	7p11.2	*EGFR*	[15]
rs4947986	7p11.2	*EGFR*	[15]

^a^Variants detected by means of GWAS in *bold*

*EGFR* is mutated and amplified in up to 70 % of primary glioblastomas [17, 18]. The EGFR family consists of four receptor tyrosine kinases: EGFR (ErbB1), Her2 (ErbB2), Her3 (ErbB3), and Her4 (ErbB4) [19]. EGFR family members are involved in many cellular processes, for example, cell proliferation and apoptosis, and are known to play a central role in development and progression in different types of cancer [20]. Deregulated EGFR signaling has been identified as a critical driver in glioblastoma tumor initiation and progression [21, 22]. Structural studies revealed that the extracellular region of EGFR is induced to homo- or heterodimerize after growth factor binding resulting in an allosteric activation of the intracellular tyrosine kinase domain [23]. However, there are multiple mechanisms causing deregulated epidermal growth factor (EGF) receptor signaling in glioblastomas, such as (i) increased EGFR abundance through gene amplification and/or increased gene translation, (ii) increased abundance of EGFR through autocrine loops, and (iii) mutations rendering the receptor constitutively active [22].

*EGFR* gene mutations are in the majority of glioblastomas accompanied by regional DNA amplification, leading to a higher number of copies of the mutated allele. The Cancer Genome Atlas (TCGA) consortium identified six somatic mutations in glioblastomas that affect EGFR protein structure, ranging from extracellular domain point mutations and deletions to deletions in the cytoplasmic tail of the receptor [24]. The aberrant exon 1–8 junction characteristic of *EGFRvIII* (Δe 2–7 vIII deletion) was highly expressed in some of the tumors investigated by TCGA. Although the biological or clinical relevance of *EGFRvIII* expression remains to be demonstrated, *EGFRvIII* expression in glioblastoma cells confers a more aggressive tumor phenotype [24, 25]. Three different C-terminal rearrangements, targeting the cytoplasmic domain of EGFR, were also detected [24]. C-terminal deletion variants have been associated with gliomagenesis in experimental rodent systems in vivo [26]. Furthermore, two relatively uncharacterized recurrent *EGFR* variants, deletions of exons 12–13 (Δe12–13) and exons 14–15 (Δe14–15) were identified by TCGA. Both Δe12–13 and Δe14–15 appear to be expressed in minor allelic fractions (<10 %), raising the question of whether they result from splicing aberration or genomic deletion [24]. This shows that somatic events in *EGFR* are common and complex and that studies of tumor heterogeneity assumes that it also is an early event as it was evident in all parts of the tumor [27].

The identified genetic risk variants for glioma are mostly mapping to intronic or intergenic parts of the genome, with no known function. To gain a better understanding of glioma etiology, the potential function of these variants requires further investigation. In this study, we investigated if there is a correlation between genetic glioma risk variants and levels of EGFR and ErbB2 in pre-diagnostic sera from glioma patients and matched controls. In particular, our investigation focused on four polymorphisms located in or close to the *EGFR* gene. The SNPs rs11979158 and rs4947979 are both located in intron 1, while rs4947986 maps to the intron-exon boundary in exon 6 or 7 of the *EGFR* gene, depending on the transcript. The fourth SNP in focus was rs2252586, which is located 107 kb downstream of *EGFR*.

## Materials and methods

### Biological samples

The study was designed as a nested case‐control study within the Janus Serum Bank of Norway [28]. Detailed descriptions of the Janus repository and the present study population have been published recently [29]. In brief, we identified 593 glioma cases by linking the Janus cohort to the Cancer Registry of Norway. Cases were diagnosed on average 15 years after blood sample collection. A total of 590 cancer-free controls were individually matched on the basis of sex, year of birth (within 15 months), county, and date of sample collection (within 4 months). All pre-diagnostic serum samples were donated between 1972 and 2004 and stored at −25 °C at the Janus repository. Most of the samples came from people in their forties undergoing health surveys of cardiovascular disease risk. Health examination samples collected between 1974 and 1978 were collected in vacutainer tubes containing 5 mg iodoacetate (*n* = 388). Around 10 % of the samples of the Janus cohort were collected from Red Cross Blood Bank donors. Red Cross blood donor samples collected between 1973 and 1975 were lyophilized (*n* = 75). All other samples underwent no special procedures after collection. The study was approved by the ethical review board at the University of Oslo, Norway.

### DNA amplification

Five-microliter aliquots of serum were transferred into 96-well microplates (Axygen, VWR, Oslo, Norway) and subjected to enzymatic amplification, as described by Ekstrøm et al. [30]. Serum aliquots were denatured and PCR master mix was added to each well, and plates were subjected to temperature cycling as described [29].

### Variant detection

Amplified 6-FAM (6-carboxyfluorescein, single isomer) labeled PCR products were analyzed by denaturant capillary electrophoresis on a MegaBACE 1000 DNA Analysis System (GE Healthcare Bio-Sciences AB, Uppsala, Sweden). The base variants were separated by cycling temperature capillary electrophoresis (CTCE), with separating temperatures. The variants were identified by co-analysis with a mutated internal standard, essentially as described by Bjørheim et al. [31]. The assay was run in a 96-well format, where a minimum of two wells per microplate were used for controls, i.e. one serum control (pool of sera from five healthy individuals) and at least one negative control without serum template. The genetic variants analyzed were selected from previous GWAS and candidate genes including rs11979158, rs2252586, rs4947979, rs4947986 (all *EGFR*), rs1412829, rs4977756 (both *CDKN2B-AS1*), rs2736100 (*TERT*), rs4295627 (*CCDC26*), rs498872 (*PHLDB1*), rs6010620 (*RTEL1*), rs78378222 (*TP53*), and rs1476278 (*PGAP3*) (Tables 3 and 4), as described by Wibom et al. [29].

### EGFR and ErbB2 serum detection

EGFR and ErbB2 serum levels were measured by a multiplex immunoassay (Meso Scale Discovery (MSD), Gaithersburg, MD, USA) according to the manufacturer’s protocol. Multi-Spot® 96-well plates from MSD were coated by the manufacturer with two different antigens to capture antibodies against EGFR and ErbB2 in human serum samples. All Multi-Spot® 96-well plates were ordered in a single lot to reduce inter-assay variation. Reagents used for the multiplex immunoassay were from MSD, if not stated separately. Recombinant human EGFR (R&D Systems Europe Ltd, Oxon, UK) and ErbB2 (eBioscience, Inc. CA, USA) were diluted in diluent 9 to prepare a calibration mix for the standard curves. Each well in the antigen-coated plates were incubated with 150 μl blocking solution C at room temperature for 1 h with shaking and washed three times with phosphate-buffered saline containing 0.05 % Tween 20 (PBS-T). Diluent 7 were added to each well and thereafter 25 μl of calibrator mix, internal controls, or samples were added to each well. The plates were sealed and incubated at room temperature for 2 h on a shaker and then washed three times with PBS-T. After that 25 μl multiplex detection antibody mix (containing Sulfo-Tag-labeled secondary antibodies against EGFR and ErbB2, Sulfo-Tag-labeled streptavidin, D-R blocker, D-M blocker, and diluent 8) was added. The plates were sealed and incubated at room temperature for 2 h on a shaker and then washed three times in PBS-T. Finally, 150 μl of MSD Read Buffer-T was added to each well. Quantification of protein concentrations was performed with MSD sector imager model no. 2400. Each analyzed assay plate included, a standard curve, case-control samples (blinded and placed in random order), and two laboratory control samples allowing to monitor inter-assay variation.

### Quality control

The quality of the genotype data was assessed by calculating call rates for both samples and genotypes. Samples that displayed a genotyping call rate of <80 % (i.e., where the genotyping had failed at >2 variants) were removed from further analyses involving genotype data. We also tested the genotype frequency distribution among controls for each SNP against the Hardy-Weinberg equilibrium (HWE) to identify potential genotyping errors. Among the serum protein abundance data, values outside mean ± 5 standard deviations were removed as outliers.

### Statistical analysis

To investigate associations between protein abundance in serum and risk of disease, we applied conditional logistic regression both to untransformed continuous abundance data and to dichotomized abundance data, where the median of the control population (EGFR 57.517 ng/ml; ErbB2 1.857 ng/ml) was used as cutoff for categorization. To investigate correlations between protein abundance in serum and a given genotype, we applied an additive linear regression model (as the samples were not matched for these analyses) and included the following covariates: sex, storage time, sample lyophilization, addition of iodoacetate, and analysis batch. We performed separate analyses for the following groups: all cases, cases with blood sample donation within 5 years to diagnosis, glioblastomas, oligodendrogliomas, astrocytomas, controls, and the entire study cohort. All analyses were performed using the R software for statistical computing (www.R-project.org).

## Results

Genotyping was successful for 1178 of 1183 samples. All included variants displayed a call rate of >90 % and a HWE *P* value >0.005, i.e., there was no indication of genotyping errors. The quantification of serum levels of EGFR and ErbB2 was successful in all but five instances. One sample failed for both EGFR and ErbB2, and the ErbB2 values from three additional samples were removed as outliers.

The characteristics of the study population are represented in Table 2. Time from blood sample collection to case diagnosis was 14.7 years (median) and ranged from 2 months to 35 years. We found no evidence for a correlation between serum levels of neither EGFR nor ErbB2 and time between sampling and diagnosis, *P* = 0.499 and *P* = 0.221 respectively. EGFR levels appeared to increase in correlation with storage time (*P* = 0.014), as assessed by linear regression adjusted for sex, analysis batch variation, addition of iodoacetate, and sample lyophilization. We noticed a similar trend for ErbB2 levels, although not significant (*P* = 0.279). By the same analysis approach, both EGFR and ErbB2 were found having higher levels in males than in females (*P* = 4.625 × 10^−6^ and *P* = 1.960 × 10^−6^ respectively).

**Table 2:** Characteristics of the study population

	Cases		Controls^a^	
	*N*	%	*N*	%
	593	100	590	100
Sex				
Female	199	33.6	198	33.6
Male	394	66.4	392	66.4
Age at sampling (years)				
18–30	53	8.9	55	9.3
31–40	220	37.1	213	36.1
41–50	295	49.8	296	50.2
51–60	8	1.3	9	1.5
61–70	16	2.7	15	2.6
71–75	1	0.2	2	0.3
Year of sampling				
1972–1985	283	47.7	280	47.5
1986–1991	246	41.5	248	42.0
1992–1997	60	10.1	58	9.8
1998–2003	4	0.7	4	0.7
Histologic subtypes				
Glioblastoma	396	66.8		
Oligodendroglioma	33	5.6		
Ependymoma	27	4.5		
Astrocytoma	94	15.9		
Other subtypes	43	7.2		
Time from sampling to diagnosis				
Mean ± SD (years)	15.2 ± 8.6			
Median serum protein level				
EGFR (ng/ml)	58.35		57.52	
ErbB2 (ng/ml)	1.95		1.86	

^a^ Controls were matched to cases on sex, age (± 15 months), and date of sample collection (± 4 months)

We assessed the associations between serum protein concentrations and disease risk (overall and by histological subtype) using both untransformed continuous abundance data and dichotomized abundance data. By dichotomizing the abundance variables, we found an association between high pre-diagnostic EGFR concentration and risk of glioblastoma (*P* = 0.008; OR = 1.58, 95 % CI = 1.13–2.22), as well as associations between high pre-diagnostic ErbB2 concentration and overall risk for glioma (*P* = 0.049; OR = 1.39, 95 % CI = 1.00–1.93) and glioblastoma (*P* = 0.017, OR = 1.63, 95 % CI = 1.09–2.44). We could not confirm these observations when investigating continuous abundance data and glioma risk (all *P* > 0.05). Median serum protein concentrations are shown for cases and controls in Table 2. Density plots were applied to visualize continuous distributions of serum protein levels for cases and controls (Supplementary figure 1).

Possible correlations between risk SNPs and serum levels of EGFR and ErbB2 were analyzed. The analyses were performed separately for overall glioma cases, glioma subtypes, control individuals, and the entire study cohort, including both cases and controls. All results are listed in Tables 3 and 4.

**Table 3:** Trends for EGFR serum levels for glioma subtypes and controls depending on genotype

SNP	Gene	Alleles	Cases	Glioma	GBM	Oligo	Astro	Cases^a^	Controls	Study-cohort
		Major│Minor						<5 years		
rs11979158	*EGFR*	**T**│C	ns	ns	ns	ns	ns	ns	ns	ns
			↑	↑	↑	↓	↓	↑	↓	↑
rs1412829	*CDKN2B*	T│**C**	ns	ns	ns	ns	ns	ns	ns	ns
			↑	↑	↑	↓	↓	↑	↓	↑
rs1476278	*PGAP3*	C│**T**	ns	ns	ns	ns	ns	ns	ns	ns
			↓	↓	↑	↓	↓	↑	↓	↓
rs2252586	*EGFR*	C|**T**	ns	ns	ns	ns	ns	ns	ns	ns
			↓	↓	↓	↑	↑	↑	↓	↓
rs2736100	*TERT*	**G**|T	ns	ns	ns	ns	ns	ns	ns	ns
			↑	↑	↑	↑	↓	↑	↑	↑
rs4295627	*CCDC26*	T|**G**	ns	ns	ns	ns	ns	**0.03**	ns	ns
			↑	↑	↑	↓	↑	**↑**	↑	↑
rs4947979	*EGFR*	**A**|G	ns	ns	ns	**0.05**	ns	ns	ns	ns
			↓	↓	↓	**↓**	↑	↑	↓	↓
rs4947986	*EGFR*	C|**T**	ns	ns	ns	ns	ns	ns	**0.009**	**0.02**
			↓	↓	↓	↓	↓	↑	**↓**	**↓**
rs4977756	*CDKN2A/2B*	A|**G**	ns	ns	ns	ns	ns	ns	ns	ns
			↑	↑	↑	↑	↓	↑	↓	↓
rs498872	*PHLDB1*	G|**A**	ns	ns	ns	ns	ns	ns	ns	ns
			↓	↑	↑	↑	↓	↓	↓	↓
rs6010620	*RTEL1*	**G**|A	ns	ns	ns	ns	ns	ns	ns	ns
			↓	↓	↓	↑	↑	↑	↓	↓
rs78378222	*TP53*	A|**C**	ns	ns	ns	nd	ns	nd	ns	ns
			↓	↓	↓		↓		↑	↓

*Arrows* indicate higher (↑) or lower (↓) serum EGFR for the risk allele (significant trends and risk allele in *bold*)

*ns* not significant, *nd* not determined, *GBM* glioblastoma, *Oligo* oligodendroglioma, *Astro* astrocytoma

^a^Cases with sample donation 0–5 years before diagnosis

**Table 4:** Trends for ErbB2 serum levels for glioma subtypes and controls depending on genotype

SNP	Gene	Alleles	Cases	Glioma	GBM	Oligo	Astro	Cases^a^	Controls	Study-cohort
		Major│Minor						<5 years		
rs11979158	*EGFR*	**T**│C	ns	ns	ns	ns	ns	ns	ns	ns
			↑	↑	↑	↑	↑	↓	↓	↑
rs1412829	*CDKN2B*	T│**C**		ns	ns	ns	ns	ns	ns	ns
			↑	↑	↑	↓	↑	↑	↑	↑
rs1476278	*PGAP3*	C|**T**	ns	ns	ns	**0.05**	**0.05**	ns	ns	ns
			↓	↓	↓	**↓**	**↓**	↑	↓	↓
rs2252586	*EGFR*	C|**T**	ns	ns	ns	ns	ns	ns	ns	ns
			↓	↓	↓	↓	↓	↓	↓	↓
rs2736100	*TERT*	**G**|T	ns	ns	ns	ns	ns	ns	ns	ns
			↑	↓	↓	↓	↑	↑	↑	↑
rs4295627	*CCDC26*	T|**G**	ns	ns	ns	ns	ns	ns	ns	ns
			↑	↑	↑	↓	↓	↑	↓	↓
rs4947979	*EGFR*	**A**|G	ns	ns	ns	ns	ns	ns	ns	ns
			↑	↑	↑	↓	↓	↑	↑	↑
rs4947986	*EGFR*	C|**T**	ns	ns	ns	ns	ns	ns	ns	ns
			↑	↑	↑	↑	↓	↑	↓	↓
rs4977756	*CDKN2A/2B*	A|**G**	ns	ns	ns	ns	ns	ns	ns	ns
			↑	↑	↑	↓	↑	↑	↑	↑
rs498872	*PHLDB1*	G|**A**	ns	ns	ns	ns	ns	**0.005**	ns	ns
			↓	↓	↓	↓	↓	**↓**	↑	↓
rs6010620	*RTEL1*	**G**|A	ns	ns	ns	ns	ns	ns	ns	ns
			↑	↑	↑	↑	↓	↑	↓	↓
rs78378222	*TP53*	A|**C**	ns	ns	ns	nd	ns	nd	ns	ns
			↓	↓	↓		↑		↓	↓

*Arrows* indicate higher (↑) or lower (↓) serum ErbB2 concentration for the risk allele (significant trends and risk allele in *bold*)

*ns* not significant, *nd* not determined, *GBM* glioblastoma, *Oligo* oligodendroglioma, *Astro* astrocytoma

^a^Cases with sample donation 0–5 years before diagnosis

Among the *EGFR* gene variants included in the study, we found a correlation between rs4947986 and decreased EGFR serum levels (*P* = 0.024), as observed in the entire study cohort. The association seemed accentuated among controls (*P* = 0.009). Similar trends were observed in major histologic subtypes investigated, albeit not statistically significant (*P* > 0.05). Another *EGFR* gene variant, rs4947979, correlated with lower EGFR concentration in oligodendroglioma cases (*P* = 0.050).

The weak correlations between risk variants of *CCDC26*, *PGAP3*, and *PHLDB1* and serum protein concentration, which were not corresponding to our predefined hypothesis, are even presented in Tables 3 and 4.

None of the described correlations of genotypes with serum protein levels were significant after adjusting for multiple testing.

## Discussion

Numerous studies have found an association between the expression of EGFR and its heterodimerization partner ErbB2 within different tumors [32–34], including gliomas [35–37]. Identifying nine glioma susceptibility loci, GWA studies have provided further information about glioma etiology, although it is unclear how these risk variants initiate pathogenic effects. This missing information offered a compelling rationale for our study: we investigated potential associations between known glioma risk variants and pre-diagnostic serum levels of EGFR and ErbB2. In addition, we evaluated the association between the risk for glioma and pre-diagnostic serum levels of EGFR and ErbB2 using both dichotomized and continuous protein abundance data to understand if chronic activation and high levels of the receptors could be a mechanism in gliomagenesis. By dichotomizing pre-diagnostic serum protein concentration according to the median of the control population, we found that both high levels of EGFR and ErbB2 were associated with glioblastoma risk. High serum concentration of ErbB2 was also associated with glioma risk overall. A chance finding cannot be ruled out, as the correlations were not evident when analyzing continuous data. It is possible, however, that specific cutoff values need to be exceeded to initiate gliomagenesis, a phenomena that has been observed for high levels of pre-diagnostic C-reactive protein and the genesis of ovarian cancer [38].

To our knowledge, pre-diagnostic serum EGFR and ErbB2 concentrations have not been studied in glioma, although it is known that elevated serum EGFR in samples taken at glioma diagnosis is associated with worse prognosis [39]. In our study, the long lag time (almost 15 years) between sample donation and glioblastoma diagnosis indicates that increased serum EGFR and ErbB2 are likely to be associated with an etiological factor more than with the effects of undiagnosed disease (i.e., reverse causation). Given that the blood-brain barrier is expected to be intact such a long time before diagnosis, it might be speculated that the observed serum protein perturbations may take their origin outside of the central nervous system (e.g., due to environmental factors). Elevated serum protein abundance could be a tumor-initiating factor as, for example, is the case for chronic inflammation due to hepatitis C which can precede liver cancer and lymphoma [40].

Both EGFR and ErbB2 serum levels displayed no detectable correlation with time between sampling and diagnosis. Because we did not have access to repeated pre-diagnostic samples, we could not dismiss concentration changes over time on the individual level. Ligand-mediated chronic activation of EGFR is necessary for gliomagenesis in mice [41], and this association might be consistent with our finding of time-stable, elevated serum EGFR and ErbB2 levels in glioblastomas. The mechanisms behind enhanced oncogenic signaling due to EGFR are extremely complex. For example, the activation of autocrine loops due to EGF ligands causes constitutive receptor activation [22]. Taking this process into account would have improved our study, but a broader screening of serum protein levels was not possible due to the limited volume of sera. However, EGFR activation and EGFR serum levels differ biologically: EGFR activation is an intracellular process, and serum levels of EGFR reflect extracellular protein concentrations.

There is high haplotype variability in the *EGFR* gene, and we analyzed a limited set of four polymorphisms, where one of them showed an association with lower serum EGFR. All four investigated genetic variants are independent of each other in terms of linkage disequilibrium. The underlying mechanisms connecting the observed association between genotype and serum levels are not clear. The polymorphism rs4947986 is located 47 bases from the boundary of exon 7 that is part of the extracellular domain II which is critical to EGFR dimerization [23, 42]. It is plausible that it is linked with a, hitherto unidentified, variant that in turn affects the protein sequence. Considering the genomic location, changes to the protein sequence or structure may have large effects on the protein’s physical properties, potentially affecting dimerization. Further understanding of the interactions between genetic variants and associations with haplotypes would require a fine mapping of the *EGFR* region in pre-diagnostic samples from other cohorts. In addition, matched blood and tumor samples with information regarding the exact type of somatic *EGFR* mutations would help classify more specific subgroups and enable identification of stronger associations.

Our study had some limitations. All genotype-phenotype associations we present here were not significant following adjustment to both the family-wise error rate (Bonferroni correction) and the false discovery rate (Benjamini-Hochberg procedure). Both procedures to adjust for multiple testing might be too stringent and imply the risk of false-negative findings because some investigated variables are not independent and due to the experimental design and the hypothesis-driven character of our study. Another limitation concerns *EGFR* variants rs4947979 and rs4947986. These variants were suggested to be associated with gliomas by a candidate gene approach that included 728 glioma individuals [15]. Recently, our group found that both SNPs may primarily be associated with prolonged survival rather than with risk in 598 glioma cases [29]. However, the two variants might be important to the mutation cascade occurring in *EGFR* during glioma tumorigenesis.

Our data provide first evidence that increased serum EGFR and ErbB2 levels can be detected in glioblastoma patients more than a decade before diagnosis, indicating that both proteins are important early in gliomagenesis. To confirm these results, we will need broader collaborations with other centers that collect longitudinal data using similar approaches. Further studies of *EGFR* and gene-environment interaction will be performed in existing and recently collected case-control studies (Amirian et al., unpublished manuscript) to understand the interaction between genetic variants and environmental factors such as asthma, allergy, and smoking.

## Electronic supplementary material

Below is the link to the electronic supplementary material.

ESM 1 Density plots comparing the continuous distributions of EGFR and ErbB2 serum levels between cases and controls for the entire study cohort as well as for glioblastoma cases and their matched controls (*dashed lines* indicate median serum protein levels; *black lines* represent controls; and *dark red lines* represent cases). (GIF 116 kb)

High resolution image (EPS 1764 kb)
