# Supplementary figures and images for: Pre-diagnostic serum levels of EGFR and ErbB2 and genetic glioma risk variants: a nested case-control study
Source: Tumour Biol. 2016 Feb 23;37(8):11065–72. doi: 10.1007/s13277-015-4742-y (PMC4999462; doi:10.1007/s13277-015-4742-y)

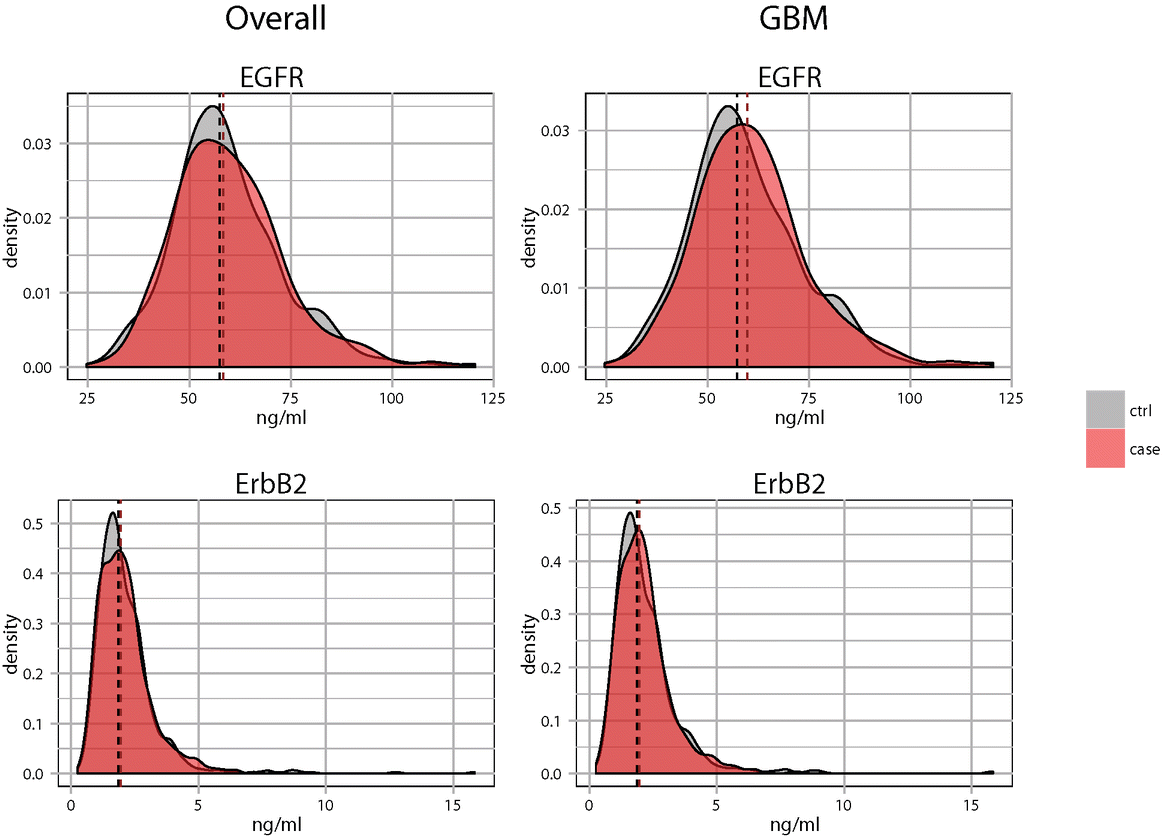

Supplement: Supplementary file 1 — Density plots comparing the continuous distributions of EGFR and ErbB2 serum levels between cases and controls for the entire study cohort as well as for glioblastoma cases and their matched controls (dashed lines indicate median serum protein levels; black lines represent controls; and dark red lines represent cases). (GIF 116 kb) [file 13277_2015_4742_Fig1_ESM.gif]
